# P-1346. Identification of aminoglycoside 6′-*N*-acetyltransferase type Ib small molecule inhibitors using mixture-based combinatorial libraries and structure-activity relationship studies

**DOI:** 10.1093/ofid/ofae631.1523

**Published:** 2025-01-29

**Authors:** Jan Sklenicka, Tung Tran, Angel J Magaña, Maria Soledad Ramirez, Haley Donow, Travis M LaVoi, Yasir Mamun, Prem Chapagain, Radleigh Santos, Clemencia Pinilla, Marc Giulianotti, Marcelo Tolmasky

**Affiliations:** California State University Fullerton, Fullerton, California; California State University Fullerton, Fullerton, California; California State University Fullerton, Fullerton, California; California State University Fullerton, Fullerton, California; Florida International University, Port St. Lucie, Florida; Florida International University, Port St. Lucie, Florida; Florida International University, Port St. Lucie, Florida; Florida International University, Port St. Lucie, Florida; Nova Southeastern University, Fort Lauderdale, Florida; University of Minnesota, Minneapolis, Minnesota; University of Minnesota, Minneapolis, Minnesota; CSUF, Fullerton, California

## Abstract

**Background:**

The aminoglycoside 6′-*N*-acetyltransferase type Ib [AAC(6’)-Ib] confers resistance to amikacin and other relevant aminoglycosides in Gram-negatives. To identify small molecule inhibitors of AAC(6’)-Ib that would overcome resistance, we utilized a strategy incorporating mixture-based combinatorial libraries, scaffold ranking, and positional scanning, followed by structure-activity relationship analysis. After identification of pyrrolidine pentamine by scaffold ranking, we isolated a compound substituted by two S-phenyl groups, an S-hydroxymethyl group, and a 3-phenylbutyl group at the R positions (compound 2700.001) (Fig 1). Herein, we describe a structure-relationship analysis of this compound.

Figure 1

**Figure:**
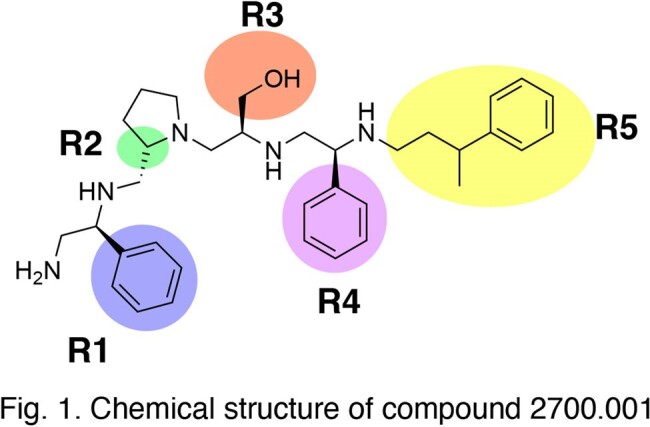

Chemical structure of compound 2700.001

**Methods:**

Initial inhibition assessment was done by measuring OD600 of *A. baumannii* A155 cultures at a single dose of compound. Checkerboard assays were done in triplicate, with each dose combination tested twice. The data were processed to eliminate any antibacterial effect exerted by the inhibitor.

**Results:**

Single substitution of functionalities at R positions along the core scaffold of compound 2700.001 showed that position R5 is the most tolerant to changes. The replacement of 3-phenylbutyl by 4-phenylbutyl resulted in compound 2700.003, equally active to 2700.001. Modifications at all other locations produced weaker inhibitors, while truncations of the scaffold resulted in inactive compounds. Testing compounds with double or triple substitutions confirmed the critical role of the S-phenyl group at position R1. No compounds with higher inhibitory activity were found. Using molecular docking, the relationship between ΔG (Kcal/mol) values for each compound and inhibitory activity was studied, with results indicating a significant correlation.

**Conclusion:**

Using mixture-based combinatorial libraries, the scaffold ranking approach, and the positional scanning strategy, we identified an inhibitor of AAC(6’)-Ib that can overcome resistance to amikacin in Gram-negatives. Furthermore, structure-activity relationship studies led to the identification of a second inhibitor, while also giving direction for further improvements to the molecule toward developing stronger inhibitors.

**Disclosures:**

**All Authors**: No reported disclosures

